# Supplementary material for: Establishment and Application of CRISPR–Cas12a-Based Recombinase Polymerase Amplification and a Lateral Flow Dipstick and Fluorescence for the Detection and Distinction of Deformed Wing Virus Types A and B
Source: Viruses. 2023 Oct 1;15(10):2041. doi: 10.3390/v15102041 (PMC10612068; doi:10.3390/v15102041)
Supplement: Supplementary file 1 [file viruses-15-02041-s001.zip › viruses-2550735-supplementary.pdf]

Table S1. Clinical sample source statistics table

Region	Sample Type	Varroa-exposed	Symptomatic
Kuandian	Beelarvae（3-day-old、5-day-old、7-day-old、9-day-old）	−	−
	Pupae	+	−
	Newly emerged bee	−	−
	Worker bee	−	+
Xinbin	Beelarvae（3-day-old、5-day-old、7-day-old、9-day-old）	−	−
	Pupae	−	−
	Worker bee	−	−
Xingcheng	Beelarvae（3-day-old、5-day-old、7-day-old、9-day-old）	−	−
	Newly emerged bee	−	−
Jinzhou	pupae	+	−
	Newly emerged bee	−	−
	Worker bee	−	+
Benxi	Pupae	+	−
	Worker bee	−	−
Songyuan	Beelarvae（3-day-old、5-day-old、7-day-old、9-day-old）	−	−
	Newly emerged bee	−	−

A

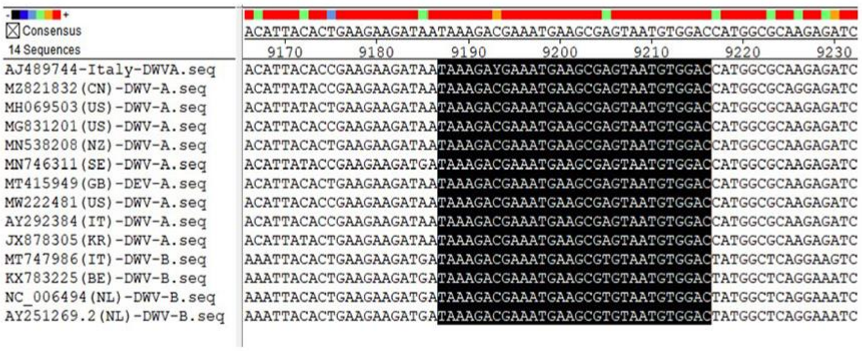

B

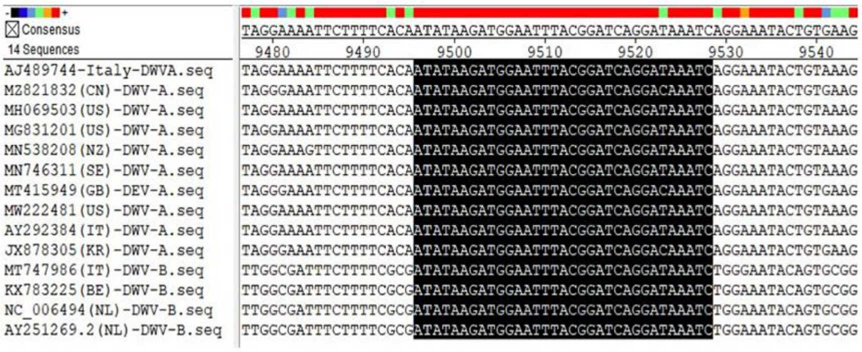

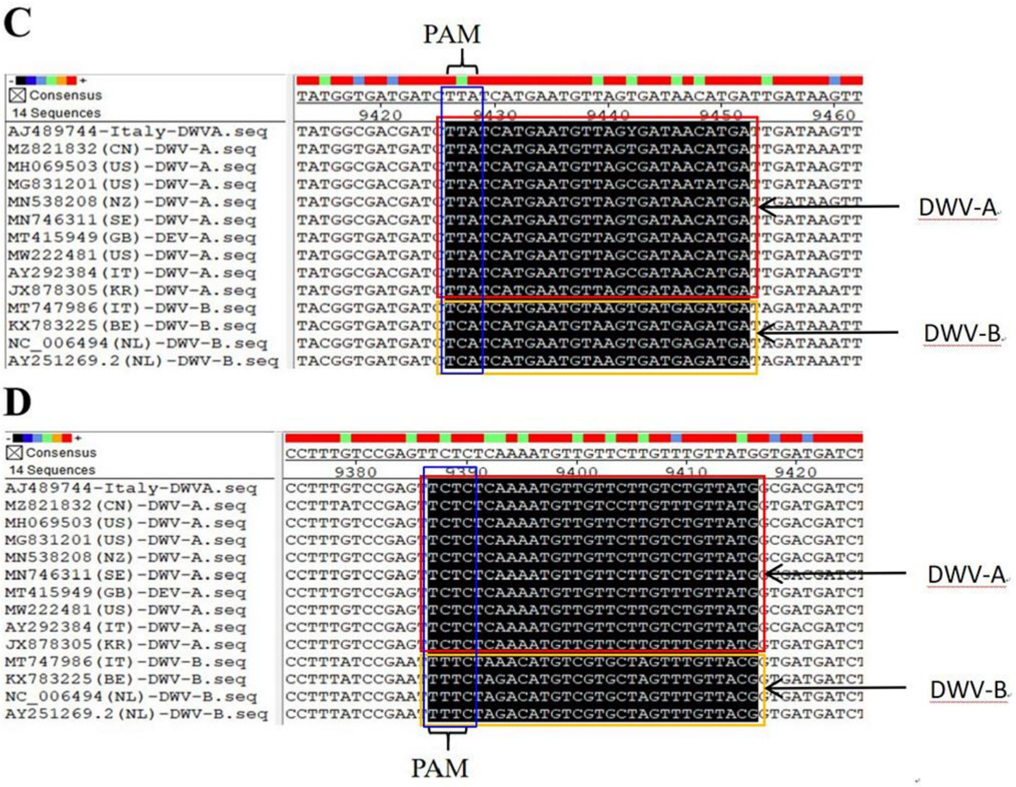

Figure S2. Alignment analysis of DWV-A and DWV-B nucleotide sequences. We selected several representative DWV virus strains to compare the target genes of DWV-A and DWV-B genes. **(A)** Forward primer and **(B)** reverse primer used for the RPA assay**. (C)** crRNA A1 and **(D)** crRNA B1 used for the CRISPR-Cas12a–FBD and CRISPR-Cas12a–LFD assay.

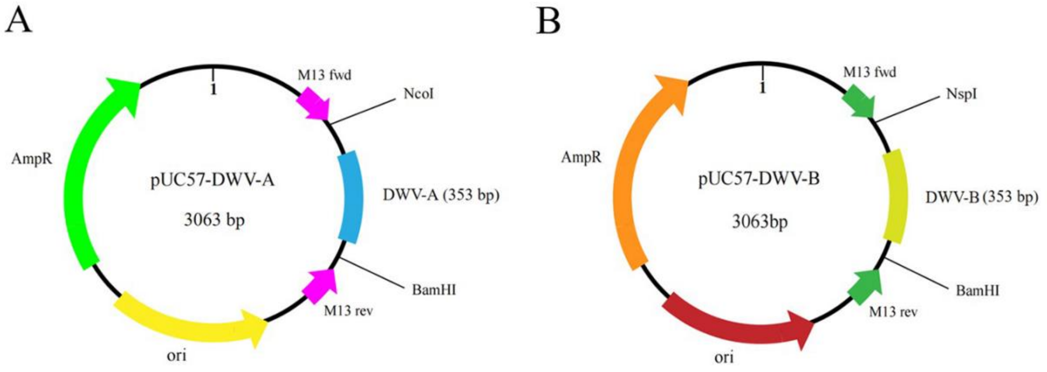

Figure S3. Recombinant plasmid construction maps for DWV-A**(A)** and DWV-B**(B).**
